# Older People Living in Long-Term Care Facilities and Mortality Rates During the COVID-19 Pandemic in Italy: Preliminary Epidemiological Data and Lessons to Learn

**DOI:** 10.3389/fpsyt.2020.586524

**Published:** 2020-10-14

**Authors:** Giovanni de Girolamo, Giuseppe Bellelli, Angelo Bianchetti, Fabrizio Starace, Orazio Zanetti, Cristina Zarbo, Rocco Micciolo

**Affiliations:** ^1^Unit of Psychiatric Epidemiology and Evaluation (UOPEV), Scientific Institute for Research, Hospitalization and Healthcare (IRCCS) Istituto Centro San Giovanni di Dio Fatebenefratelli, Brescia, Italy; ^2^School of Medicine and Surgery, University of Milan Bicocca, Milan, Italy; ^3^Head of the Acute Geriatric Unit, San Gerardo Hospital, Monza, Italy; ^4^Department of Medicine and Rehabilitation, S. Anna Hospital, Brescia, Italy; ^5^Department of Mental Health and Addiction, Local Health Authority of Modena, Modena, Italy; ^6^Operative Unit (UO) Alzheimer-Memory Clinic, Scientific Institute for Research, Hospitalization and Healthcare (IRCCS) Istituto Centro San Giovanni di Dio Fatebenefratelli, Brescia, Italy; ^7^Department of Psychology and Cognitive Sciences, University of Trento, Trento, Italy

**Keywords:** long-term care facilities, older people, mortality rate, COVID-19, risk factors

## Abstract

**Background:** Long-Term Care Facilities (LTCF) in Italy have been particularly affected by the COVID-19 pandemic, especially in terms of mortality rates of older residents. However, it is still unclear the actual extent of this situation. The aim of this manuscript is to assess the extent of mortality rates of older adults in LTCF during the pandemic across different regions of Italy, compared to the previous years and to older general population not resident in LTCF.

**Methods:** We extracted and analyzed data collected by three Italian institutions (i.e., Italian Statistician Institute ISTAT, Italian N.I.H, Milan Health Unit) about the number of deaths among older people living in the community and among LTCF residents during the pandemic and the previous years. We also compared the observed mortality rate among LTCF residents in each Italian Region with the corresponding expected number of deaths of the general older adult population to obtain an observed/expected ratio (O/E ratio).

**Results:** During the pandemic, about 8.5% (*N* = 6,797) of Italian older adults residents in LTCF died. Findings resulting from the O/E ratio suggest that LTCF residents (in particular in the Lombardy Region) show higher mortality rates when compared to expected values of mortality rates among the older general population living in the community. Furthermore, we found that the risk of death among LTCF residents increased about 4 times during the pandemic when compared to the previous years.

**Conclusions:** Mortality rates in LTCF were high during the pandemic, especially in Lombardy. Possible causes of higher mortality rates in LTCF and suggestions for specific targeted interventions are discussed.

## Introduction

Italy is one of the countries most violently affected by the Coronavirus-Disease 19 (COVID-19) pandemic and outbreak. As of July 22nd, 244,708 persons (median age 61 years) were known to have contracted the infection and 34,126 (13.9%) died (1). A recent review on COVID-19 pandemic highlights the urgent need to give appropriate attention to the more sensitive population groups, including children, healthcare workers, and older individuals (2). In particular, older adults deserve specific attention as they are at higher risk of both contracting COVID-19 (3, 4) and of negative prognosis or death due to it (3–8).

The higher predisposition of older adults to COVID-19 and their negative prognosis seem to be due to preexisting chronic comorbidities [e.g., hypertension, diabetes, cardiovascolar diseases; (8)] and to an higher likelihood of developing clinical complications after having contracted the virus [probably due to higher predisposition to contract bacteric infection, and to changes in pulmonary anatomy; (7)]. A specific vulnerable subgroup is represented by older adults with dementia, since they may have cognitive deficits which may limit their understanding and memory of safeguard procedures, which may lead, in turn, to an higher risk of infection (9).

However, the link between age, disability, and COVID-19 risk of mortality is still unclear and needs further clarification. Indeed, despite the increasing evidence about the role of age in affecting the risk of contracting the COVID-19 virus and mortality risk, a recent study, investigating the association between frailty and in-hospital mortality due to COVID-19 in the UK and Italy, found that disease outcomes of older adults were better predicted by frailty than either age or comorbidity (10). Long-Term Care Facilities (LTCF) for older people have been particularly affected by the pandemic in terms of number of infections and mortality rates (11). Indeed, COVID-19 related deaths in LTCF residents represented 30–60% of all COVID-19-related deaths in many European countries (11). Despite the public health relevance of this issue, only a few articles have specifically addressed the problem of COVID-19 among LTCF residents (12–14), and so far none of them has been conducted in Italy. Thus, the toll of deaths of older adults in these facilities still remains to be clarified.

The aim of this manuscript is to analyze the mortality rates of older adults in LTCF across different regions of Italy, compared to older general population not resident in LTCF during the COVID-19 outbreak; we will also explore mortality rates of Milan LTCF residents during the outbreak compared to the previous 4 years. This analysis may provide important insights to prevent, control and mitigate future pandemics within LTCF, to allocate appropriate resources (in terms of manpower and equipments) to allow these facilities control and mitigation, to identify specific at risk populations for psychological suffering (e.g., healthcare workers in LTCF, relatives of patients who died) during the post-pandemic phase, and to target specific psychological and medical interventions.

## Methods

We extracted, analyzed, and compared data collected by the Italian National Health Institute (N.I.H.), the National Statistical Institute (ISTAT), and the Milan Health Unit.

Data about deaths among LTCF at the time of COVID-19 has been collected by the Italian N.I.H. through a brief online survey (15), started on March 24th, targeting 3,420 public or private LTCF (reimbursed by the National Health Institute or by municipalities) included in the “*Dementia Registry*.” The survey was conducted with a 29-item questionnaire aimed at assessing the consequences of pandemic and the procedures and behaviors adopted to reduce the risk of COVID-19 contagion. The survey was firstly e-mailed to the Directors of facilities and followed then to additional phone calls (~3,042) to solicitate a reply. LTCF located in Basilicata and Valle d'Aosta regions did not reply to the N.I.H. survey and, for this reason, were excluded from the analyses.

As of April 14th, 2020, 3,276 LTCF (92.6% of the total) have been contacted, and 1,082 answered, that is 33.0% of the total sample. In the 1,082 participating LTCF (5 did not report this information), there were 80,131 residents as of February 1st, 2020, with an average of 74 residents for each facility (range 7–632). Lombardy LTCF were hosting the largest number of both residents (*N* = 23,594) and LTCF (*N* = 678). The ratio between the number of LFCT and total residents provide us the average number of number of beds of each facilities in Lombardy (*N* = 35).

Mortality rates of LTCF residents have been compared to mortality rates of specific age groups of the general population, regularly collected by the National Statistical Institute (ISTAT) and freely accessible on the ISTAT website (16).

Furthermore, we extracted data from a recent report of the Milan Health Unit (17) reporting data on mortality rates among about 16,000 residents (aged >70) of 162 LTCF located in the Province of Milan during the first 4 months of 2020 compared to the previous 4 years. The report also compared mortality rates of LTCF residents with the general population aged over 70 years, living in the same catchment area.

### Statistical Analysis

Since age-specific mortality rates in LTCF surveyed in the N.I.H. report (15) were not available, we accessed a ISTAT report (18), showing the age structure of the overall population resident in LTCF to estimate residents' mean age: that is, 73 years considering the midpoint of each age category or 77 years as the oldest possible mean age, considering the upper end of each age category.

Based on this estimate, we compared national mortality rates for each Italian Region in the age categories 70–74 and 75–79 to the number of LTCF residents in the corresponding Regions, to obtain the corresponding expected number of deaths. Expected deaths in each Italian Region were calculated multiplying the number of residents by the age-specific mortality rates of the Italian population of the same Region (75–79 years and 70–74 years columns). Then, we compared the observed with the expected number of deaths to obtain an Observed/Expected ratio (O/E ratio).

Finally, we extracted data reported in the Milan Health Unit report (17) to compare mortality rates recorded in the first 4 months of 2020 in 162 LTCF with average mortality rates recorded in the same facilities in the 4 previous years (2016–2019) during the same period (i.e., from 1st January to 28th April).

## Results

**Figure 1:**
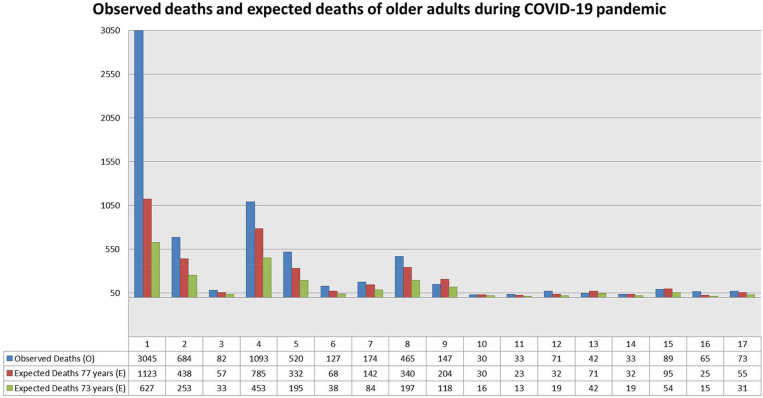
Observed deaths in LTCF residents and expected deaths at 77 and 73 years. Old age groups of the Italian general population by Regions. For the numbering of different Regions, see numbers in Table 1.

The N.I.H. survey shows that during the pandemic, 8.5% (*N* = 6,797) of Italian older adults residents in LTCF died. Table 1 shows the number of total deaths recorded among the 1,082 LTCF participants to the N.I.H. survey, and the corresponding mortality rates per 100 residents over 2 months (65 days) starting from February, 1st. As shown in Table 1, there is a marked difference in mortality rates between different Regions, with Lombardy showing the highest rate (12.9) and others, including neighboring Regions, showing remarkably lower rates; the mean of the rates shown in the 4th column of Table 1 is 7.0 and the standard deviation is 2.5. Table 1 also shows the number of expected deaths in the same period according to two hypothesized residents' mean age: 77 years and 73 years. If we consider a residents' mean age of 77 years, therefore applying the 75–79 age-specific rates of the national population (5th column of Table 1), the ratios of observed to expected deaths (O/E) for all Regions (but four, Lazio, Umbria, Calabria and Puglia) were >1, suggesting an higher mortality among LTCF residents. The highest ratio was found in Lombardy: in this Region, observed deaths were about three times those expected; in Sardegna we found a similar value (2.6), while in Abruzzo-Molise the O/E ratio was 2.1. On the other hand, assuming a mean age of 73 years for expected deaths (therefore applying the rates shown in the 8th column of Table 1), this leads to much higher values of O/E ratios; in this case, deaths observed in subjects living in Lombardy LTCF were almost five times than those expected, and in all Regions (but two, Lazio and Calabria) the O/E ratio was 1.5 or greater. Figure 1 provides a graphical overview of the observed and expected deaths in each Italian region.

**Table 1:** Number of deaths recorded in LTCF, mortality rates in older population groups, expected deaths, and Observed/Expected ratio.

	**Living in nursing homes**			**Mean age: 77 years**			**Mean age: 73 years**		
Region^*^	Residents^**^	**Observed deaths (O)**	**Mortality rate (per 100 residents)**	**ISTAT 75–79 mortality rate (%)**	**Expected deaths (E)**	**O/E**	**ISTAT 70–74 mortality rate (%)**	**Expected deaths (E)**	**O/E**
1. Lombardy	23,594	3,045	12.9	4.76	1,123	2.71	2.66	627	4.85
2. Piemonte	8,729	684	7.8	5.02	438	1.56	2.90	253	2.70
3. Liguria	1,128	82	7.3	5.05	57	1.44	2.90	33	2.51
4. Veneto	16,815	1,093	6.5	4.67	785	1.39	2.69	453	2.41
5. Emilia-Romagna	7,137	520	7.3	4.65	332	1.57	2.73	195	2.67
6. Trentino Alto Adige	1,538	127	8.3	4.43	68	1.87	2.49	38	3.32
7. Friuli V.G.	2,936	174	5.9	4.85	142	1.22	2.86	84	2.07
8. Tuscany	7,399	465	6.3	4.59	340	1.37	2.66	197	2.36
9. Lazio	3,913	147	3.8	5.21	204	0.72	3.02	118	1.25
10. Umbria	664	30	4.5	4.53	30	1.00	2.46	16	1.84
11. Marche	511	33	6.5	4.50	23	1.43	2.45	13	2.63
12. Abruzzo e Molise	638	71	11.1	4.97	32	2.24	2.94	19	3.78
13. Calabria	1,309	42	3,2	5,43	71	0,59	3,22	42	1,00
14. Campania	512	33	6.4	6.18	32	1.04	3.66	19	1.76
15. Puglia	1,866	89	4.8	5.11	95	0.93	2.89	54	1.65
16. Sardegna	526	65	12.4	4.84	25	2.55	2.87	15	4.31
17. Sicilia	916	73	8.0	5.97	55	1.34	3.36	31	2.37

* *Basilicata and Valle d'Aosta did not provided reply to the N.I.H. survey and were excluded from the analyses. Data from Molise were aggregated with those of the neighboring region Abruzzo. Data from the two autonomous provinces of Bolzano and Trento were aggregated and presented as “Trentino Alto Adige”*.

** *Residents up to February 1st, 2020 and new admissions as from March 1st, 2020*.

**Figure 2:**
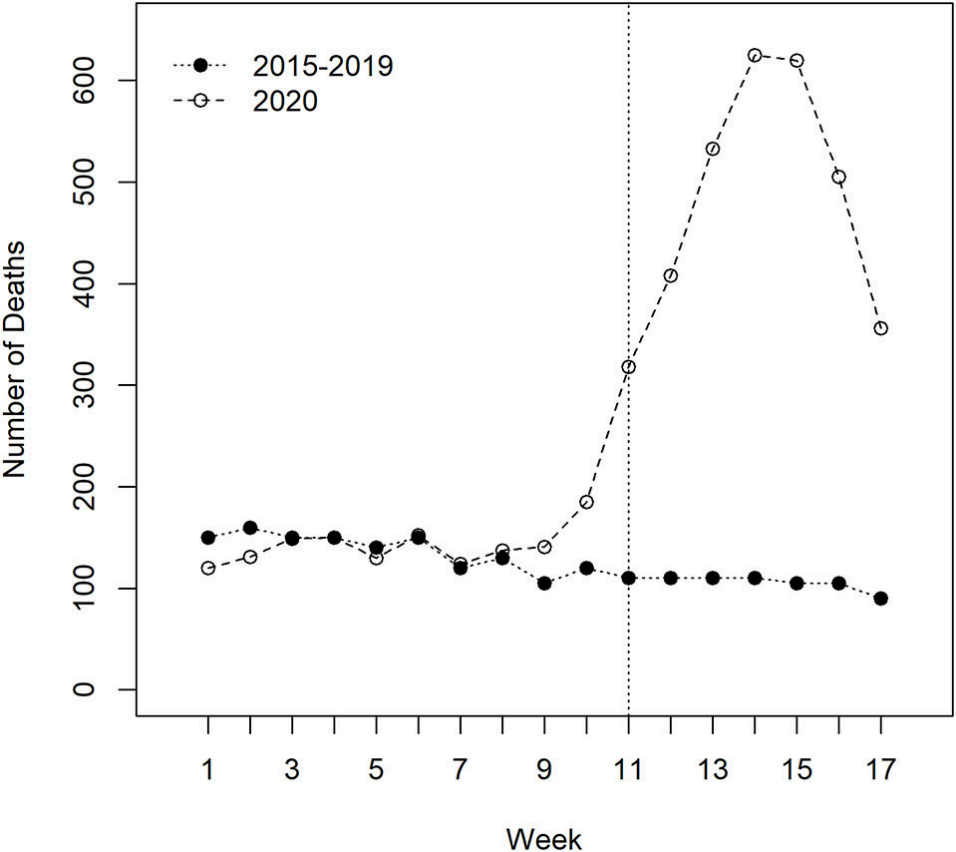
Weekly number of deaths between residents in Long-Term Care Facilities of the “ATS Città Metropolitana di Milano” (Health Protection Agency of Milan) between 1/1/2020 and 28/4/2020 compared with the mean number of deaths of residents during the corresponding periods of 2015–2019. Week number 1 starts on January 1 and ends on January 7, and so on up to week number 17, which starts on April 22 and ends with April 28. A clear increase in the number of deaths is evident from week number 11 (which starts on March 11). The peak was observed during the week number 14 (which starts on April 1). Source: “Valutazione degli eccessi di mortalità nel corso dell'epidemia Covid-19 nei residenti delle Rsa” ATS di Milano, 11th June 2020.

Figure 2 is based on data collected by the Milan Health Unit (17); it compares daily mortality rates among LTCF residents from January 1, 2020 up to April 30, 2020 with mean daily mortality rates for years 2016–2019 for all subjects aged 70 and older living in the same facilities. It is noticeable the excess of deaths after March 1, 2020: in the 2 months March-April 2020 there has been a 4-fold excess of deaths compared to the same period of the previous years, with a peak in April. Overall there has been an excess of over 2,550 deaths in the period January-April 2020, and most of this excess is concentrated in the period March 1—April 30. The increase in overall risk from January 1 to April 30 was a value of 2, while in the period from March 1 to April 30 (when the risk excess was also visible in the general population) the increase in risk of death increases to ~4 times the reference mortality values for 2016–2019.

## Discussion

In this epidemiological report comparing mortality rates of Italian community-dwelling older people with those of LTCF residents during the COVID-19 pandemic we found that during the COVID-19 pandemic, mortality in LTCF was higher than expected, using the general population as the reference group; interestingly, the excess of mortality we found in the N.I.H. sample (which was a convenience sample, including only 33% of the initial target sample) was very similar to the rate found by the Milan Health Unit including all 162 LTCF located in the Milan City, with a 2-fold increase in the period January-April and a 4-fold increase in the period March-April compared to the previous years. Furthermore, according to ISTAT (19), in March and April 2020 the analysis of general mortality rates leads to an estimated number of 45,186 deaths in excess compared to the same period of 2015–2019; among them, 28,282 (63%) might be considered due to COVID-19 according to reports of the Integrated Surveillance system. Mortality rates were different across LTCF in various Regions, with Lombardy being the Region with the highest mortality rate. Moreover, mortality rates among LTCF residents in the province of Milan (one of the areas most violently hit by the pandemic) were much higher compared to the mean rates found in the previous 4 years in the same facilities.

### Why Are Residents in LTCF at Higher Risk of Death During the COVID-19 Pandemic?

Data regarding mortality rates in LTCF is noteworthy. According to the Epidemiological Office of the Lombardy Region (20), the official annual mortality rate in Lombardy LTCF was 21.0 deaths per 100 residents both in 2017 and in 2018; this rate equals to 3.7 deaths per 100 residents in a 65-day period (the same timespan covered by the N.I.H. survey). At odds with these findings, we found a mortality rate of 12.9 per 100 LTCF residents in Lombardy in the recent COVID-19 survey, that is about 4 times as those recorded in 2017 and in 2018. Very similar findings have been found in the Milan Health Unit epidemiological study. This marked discrepancy among mortality rates clearly suggests that the COVID-19 pandemic is responsible for the increased mortality rate. In this perspective, the excess of mortality in LTCF compared to the general population cannot be explained only by a higher proportion of chronic diseases among LTCF residents. In fact, multimorbidity, geriatric syndromes, dementia, frailty, malnutrition, and disability, despite being disproportionately more common among LTCF residents, should not be considered as the leading cause of death, but, as most, as predisposing factors. Among them, frailty has been recently recognized to play a key role in heightening the risk of death due to COVID-19, more than age or comorbidity (10).

Moreover, the Milan Health Unit study shows that the mortality excess was visible also comparing residents during the 2020 pandemic with residents of the same facilities in previous years.

There are several tentative explanations for the increased mortality among older people in LTCF. Acute disorders in older people do not always present with the typical symptoms found among younger people, indirectly suggesting that the recognition of COVID-19 infections in LTCF residents might be challenging (21). This represents an important problem for the infection control. Moreover, standardized protocols for the evaluation and management of COVID-19 among LTCF residents have been missing for many weeks since the start of the pandemic, therefore leading to wide variations in the management of older patients living in LTCF. The lack of imaging facilities, the shortage of laboratory facilities and consultants (such as specialists in infectious diseases and respiratory care) in LTCF has represented a further obstacle to the safe management of infected patients. Moreover, not all LTCF are staffed with dedicated physicians on site and geriatricians, among facility physicians, are an exception rather than the rule (22).

Prevention of COVID-19 transmission was likely to be another factor affecting mortality rates in LTCF. Though we do not have official data about this, laboratory tests have been routinely available nor for LTCF residents and health care personnels, making difficult the separation between COVID-19 positive and negative subjects, and probably contributing to spread the infection. The shortage of Personal Protective Equipments (PPE) for physicians, nurses and health-care workers, repeatedly broadcasted, may have been an additional risk factor.

### Variation in Mortality Rates Across Different Regions

The huge difference in LTCF mortality rates among Italian Regions also deserves a comment. Lombardy was the Region that paid the highest toll of deaths in these facilities. It is possible that specific healthcare policies in this Region may have, at least partially, contributed. In the first days after the development of the pandemic, hospitals were overcrowded and some patients were transferred to LTCF, with obvious consequences for the risk of infection spread. Another possible explanation may have to do with the virulence of the COVID-19 virus. A recent study has shown that, soon after starting of the pandemic, the virus has mutated and that European, North American and Asian strains coexisted, each of them characterized by a different mutation pattern (23). Accordingly, it may be hypothesized that some genetic mutations, if present, might be correlated with different COVID-19 related mortality rates.

In addition, the number of LTCF beds per capita, controlling for the proportion of adults aged 75 and older and population density, has been recently found to be significantly associated with COVID-19 mortality rates (24). These findings suggest that structural features of LTCF might have affected the impact of the infection on mortality rates. In Lombardy the average number of LTCF beds in each facility is about 35, and this number is higher compared to other Italian Regions (e.g., Emilia-Romagna region has a mean of 20 beds for each LTCF): we may therefore suppose that the higher likelihood of death in Lombardy LTCF may be explained, among other reasons, also by the higher concentration of older people, with heightened risks of spread, and heavier problems in patients' management.

## Conclusions and Implications

These findings provide some insights for preventing, controlling, and mitigating future possible epidemics within LTCF, for better allocating specific funds in the case of emergencies, for allowing facilities most hit to relieve after the pandemic, and for identifying specific at-risk populations groups.

A priority is to ensure an easy and rapid access to appropriate testing for the identification of COVID-19 cases among LTCF residents and healthcare workers. Another priority is to make available standardized, clear procedures for the consistent management of epidemics in LTCF. Unfortunately, both these points have not been achieved so far (25).

It is also necessary to support health personnel and rescuers, often highly distressed. This need has been largely neglected so far and needs a proper reflection. Specific targeted psychological interventions should be oriented to healthcare staffs of LTCF, relatives of older people who died because of COVID-19, as well as to other older people who survived to COVID-19 and are still living in LTCF. Exposure to complex grief for these vulnerable groups should be taken into consideration and specifically targeted. The analyses presented here should be considered preliminary and largely descriptive. The N.I.H. survey on LTCF is still ongoing, and results reported here come from ~1/3 of the total sample. There are several limitations which should be taken into account. Firstly, age-specific mortality rates in LTCF were not reported in the N.I.H. report and were based on ISTAT data. Furthermore, no information about previous health conditions, comorbidity or causes of death of LTCF residents were included in the N.I.H. report. Data on age-specific mortality rates and comorbidities are available only for the general older population. Age-specific mortality rates among older subjects COVID-19 positive were 9.8% among 60–69 years old, 24.2% among 70–79 years old, 29.0% among 80–89 years old and 24.7% among those aged 90 years old and over (26). Furthermore, in an analysis of 4,942 death certificates (based on 31,573 reports received by the Integrated National Surveillance System for COVID-19 as of 25 May 2020), ISTAT (27) has estimated that COVID-19 was the direct cause of death in 89% of SARS-CoV-2 positive deaths. The proportion of deaths in which COVID-19 was the direct cause of death varies according to age, reaching a proportion as high as 92% among the 60–69 year-old people (with quite similar values among older classes: 90% among 70–79 year-old and 88% among 80 and older). The most frequent contributory causes of death associated with COVID-19 were hypertension (18% of deaths), diabetes mellitus (16%), ischemic heart disease (13%), and cancer (12%).

While we acknowledge that data regarding age-specific mortality, comorbidities and cause of death of older residents would allow a more specific and deep assessment of the impact of COVID-19 pandemic in Italian LTCF, unfortunately the lack of this data in the original N.I.H. report makes additional analyses or conclusions impossible. We may only assume that LCTF residents are older (>70 years old), are not totally autonomous and exhibit comorbidities which may increase the likelihood of both contracting the virus and having a negative prognosis. However, the comparison of mortality rates in LTCF during the pandemic with those in previous years (2016–2019) allows us to assume that the significant increase in mortality rates in LTCF during the pandemic may have been triggered by the COVID-19, independent of pre-existing specific comorbidities. Future targeted investigations should address these limitations in order to increase our knowledge in this area.

Despite the above mentioned limitations, available data is significant as shows a trend and points to the urgent need of appropriate measures to be taken to stop, or reduce, the increased mortality rates among very frail subjects, such as those living in LTCF. The high mortality rates observed among LTCF residents during the COVID-19 pandemic has triggered a debate about the overall organization and management of these facilities, about patient-staff ratios, about healthcare personnel's skills and, more in general, about resources allocated by the Italian healthcare system in this sector. Even if a unique model of LCTF organization and management cannot be implemented in all Italian Regions for administrative and political reasons, it is clear that it is necessary to allocate more resources for the care of frail patients, especially in LTCF (28). The “hospital-centered” health system in which the LCTF acts as a passive actor has proven to be totally unsuitable for the proper management of emergencies such as the COVID-19 pandemic (29). For this reason, models of care in LCTFs will need to be reformulated in a more personalized way, while the role of primary care and long-term care will be more and more important in future health systems. It would be desirable that more geriatricians will be employed in LTCFs and that continuing geriatric education will become a mandatory requisite for all LCTF healthcare staff.

## Data Availability Statement

Publicly available datasets were analyzed in this study. Data are available on request to the corresponding author.

## Ethics Statement

Ethical review and approval was not required for the study on human participants in accordance with the local legislation and institutional requirements. Written informed consent for participation was not required for this study in accordance with the national legislation and the institutional requirements.

## Author Contributions

GG had the initial idea and wrote the first draft. GG and RM collected the data. RM analyzed the data. All authors carefully reviewed, discussed, and contributed to various draft of the manuscript. All authors approved the final manuscript.

## Conflict of Interest

The authors declare that the research was conducted in the absence of any commercial or financial relationships that could be construed as a potential conflict of interest.
